# Bibliographical Notices

**Published:** 1840

**Authors:** 


### Notices of some New Works.

I. Traité Philosophique de Medecine Pratique. Par *A. N. Gendrin*, D.M. 2 tomes. Paris, 1838-9. *Bailliere.*

II. Traité Clinique du Rheumatisme Articulaire. Par *J. Bouillaud.* Paris, 1840. *Bailliere.*

III. Du Traitement Moral de la Folie. Par *F. Leuret.* Paris, 1840. *Bailliere.*

In the present Number we cannot do more than merely announce the titles and the general scope of the above works recently published in Paris: next quarter we shall submit them to a closer examination, and extract whatever we may find novel and valuable in their contents.

The first on the list is announced to be "*un ouvrage dogmatique*, which is to bring together and systematise all the departments of medicine which are specially applicable to the knowledge and treatment of diseases." Two volumes of the work have already appeared, and other two are promised. It would not be altogether fair to anticipate the result of our analytic review by giving an opinion at present on the merits of this "Philosophical Treatise," and we must therefore leave our readers in suspense as to the verdict that awaits the appellant.

Suffice it to say that M. *Gendrin* is one of the physicians of the La Pitié hospital in Paris, and is the author of several esteemed works. In 1823 he published Researches on the Nature and Proximate Cause of Fevers in two volumes; in 1827, an Anatomical History of Inflammations, (founded we believe, on a translation of the excellent and now rare work of Dr. *Thomson* of Edinburgh); in 1832, An Account of the Epidemic Cholera of Paris; and in 1835, a Translation with notes of Dr. *Abercrombie's* Classical Treatise on the Diseases of the Brain and Spinal Cord. We have reason therefore to expect that M. *Gendrin* may show himself better acquainted with the medical literature of this country than most of the French authors in the present day, who in this respect are most egregiously ignorant, and are in consequence often led to announce as discoveries what have been taught for the last quarter of a century in every respectable medical class-room in Britain.

With respect to M. *Bouillaud's* new work, or rather new edition of his former work "Researches on Articular Rheumatism, &c." published in 1836, the readers of the Medico-Chirurgical Review need scarcely be told what is the character of its contents and reasoning. Who has not heard of the "nouvelle formule" of practising bleeding *coup sur coup*, by which M. *Bouillaud* utterly extinguishes (*jugule,*

to use his own word) all inflammatory diseases? He is certainly the most sanguinary doctor that we have ever encountered—sanguinary, be it remembered, towards his patients, and not to us gentle reviewers. Like *Murat,* he may be well called "le grand *sabreur;*" we have only to substitute the doctor's lancet for the swordsman's scimitar, and the comparison is exact. There is something withal very amusing in our author's writings; quick and fluent in thought and language, he is as bold, and confident in the closet as in the wards of La Charité Hospital.

Hear what he says of certain of his *confreres* who have presumed to disagree with him in his favourite dogmata:—

"In medicine there are men who pride themselves in never learning and never forgetting any thing; unfortunates who have not received from Heaven the noble spirit of advance. They may be compared to those in whom religion is utterly wanting; both are fatally condemned to final impenitence. They have eyes but they will not see, because they close them, ears but they will not hear because they shut them. Praying for better dispositions to them, we must leave to others the task of attempting their recovery, if this be possible, and if their malady be not such as divine grace alone can effectually relieve."

*Leuret's* work is an interesting one, and being founded on the author's extensive experience at the Bicetre Hospital commands attention. "The object I have in view," says he, "is to establish the truth of the following three propositions:—

1. If it be true that insanity depends on an alteration or morbid condition of the encephalon, we are completely ignorant in what this alteration consists.

2. The moral treatment of the insane, as usually practised, is considered only as an auxiliary of the physical treatment.

3. In the insane, the intellect and passions cannot be brought back to their healthy type or standard without the aid of moral treatment; and this mode of treatment is the only one which has a direct influence on the symptoms of insanity.

M. *Leuret* is of opinion that in the management of mental disorders, too much is attempted to be done by physical or medical remedies, and far too little by moral means, in the way of persuasion, remonstrance, and, if necessary, of compulsion, and even chastisement.

---

MANUEL PRATIQUE DE MAGNETISME ANIMAL. Exposition Méthodique des Procédés employés pour produire les Phénomènes Magnétiques et leur Application a l'Etude et au Traitement des Maladies. Par *Alph. Teste,* Docteur en Médecine de la Faculté de Paris, Membre de plusieurs Sociétés Savantes. Paris, 1840.

We had scarcely expected this. We really had thought that Animal Magnetism had got, for the present, a knock-down blow. Not at all. Dr. Teste has been applied to by three publishers for a Manual of the *Art.* He justly observes that nothing can be more conclusive on its popularity. Your booksellers are the real barometers of public opinion. They have in their ledgers the real statistics of what takes.

And so animal magnetism is at a premium, and a manual is in print. M. Teste asks himself what the faculty, the magnetisers, and the public will say of it? Now it may be laid down as an axiom, that a man never asks himself a question in print, that he does not know before-hand he can answer: accordingly Dr. Teste makes no bones of a reply to his interrogatory. The faculty will abuse it—the magnetisers will hold their tongues about it (the old story—"two of a trade,"&c and the public will patronise it. Well done, Dr. Teste.

The Doctor asks himself another question, which, though certainly a poser, he disposes of rather cleverly. What is magnetism? says the Doctor to himself —buy my book, and you will know, says the Doctor to the reader.

The aim he proposes, and it is reasonable enough, is to teach magnetism, to disseminate its elements through all classes of society, and to make humanity understand the immense advantages that said humanity will draw from it. "God grant," quoth Dr. Teste, "I may succeed." Amen say we.

Dr. Teste is a magnetiser of no small calibre. He does all the orthodox miracles—transposes the senses—divines, &c. as well as even the Okeys.* He relates some capital cases, such as a perfectly authentic one of a lady who read through the lid of a box *well* locked—"the possible is immense," and, in the reading, proved it.

Dr. Teste concludes with a chapter on the necessity of magnetisers being *moral* men. We think that this is most laudably candid, and it is very true. Only conceive the power of a magnetiser, and it will be evident how virtuous he ought to be. A man who can see with his fingers, who can lay bare the very soul itself, and reveal the secret thoughts of men and women should be a very pattern of the moral excellencies. Shakspeare, with that knowledge of human nature for which he is always so conspicuous, and with that physiological acumen which he not unfrequently exhibits, has hit off this point exactly. For does not Hotspur say to Owen Glendower, that first and greatest of magnetisers, who could call spirits from the vasty deep—

"Tell truth and shame the devil."

It would have been well if this pithy advice had been taken by the philosophical sect that has sprung from Owen's loins. But even now it is not too late to repent. We admit that it is rather hard to ask the magnetisers to tell nothing but the truth, for it would leave them very bare in the world. Still the prejudice goes in favour of veracity.

We must not however enter on this fascinating subject. We conclude, then, by recommending this Manual to all who believe in transposition of the senses, who are of opinion that they can read through the lid of a box, who are conscious that they know the thoughts of others, who can predict the day of death of a magnetised *Jarvey*, provided he is not in the interim run over†—and, finally, to use the expressive and significant language of Mayo, just then in high *rapport*, who aspire to the awful power of "dislocating the soul from her usual tenement, and making her hold on to unaccustomed points and corners of the frame."

---

THE RETROSPECTIVE ADDRESS, DELIVERED AT THE SEVENTH ANNIVERSARY MEETING OF THE PROVINCIAL MEDICAL AND SURGICAL ASSOCIATION, HELD AT LIVERPOOL, July 24th and 25th, 1839. By *John Addington Symonds*, M.D., Senior Physician to the Bristol Hospital, Lecturer on the Practice of Medicine, &c. &c. Worcester, 1840.

Of Dr. Symonds we have so frequently had occasion to speak highly that, we believe, we need not introduce him formally to our readers. The present Address does not derogate from his reputation—it breathes the same spirit of cautious generalization and correct judgment, and evinces the same liberal acquaintance with the literature of his profession as ever.

---

* By the way, where are these marvellous maids? "The oracles are dumb."

† A magnetic fact.

We quote a succinct, yet sufficient summary of the latest experiments on the glosso-pharyngeal and the lingual nerves. They leave our information very near what we conceive to be the truth.

The part, says Dr. Symonds, which the glosso-pharyngæal takes in deglutition, is to convey impressions to the medulla oblongata, together with the branches of the fifth pair distributed on the fauces, and also with the superior laryngæals: it is not a motor nerve. The motive influence in this function is transmitted along the pharyngæal branches of the vagus, along the hypoglossal, the fifth pair, the portio dura, and the descendens noni. The superior laryngæals are mainly nerves of sensation, having a very few motor nerves distributed to the cricothyroid muscles. The inferior laryngæals are principally motor; they supply all the muscles attached to the arytænoid cartilages, and perhaps to the muscular fibres of the trachea: they contain a few sensitive filaments distributed over the trachea and pharynx, and a very few over the larynx. Contraction of the glottis is evidently a reflex action; the superior laryngæals being the afferent nerves, and the inferior laryngæals the efferent. In the account of the effects of division of the vagi in the middle of the neck, i. e., above the origin of the recurrents, there is a curious observation bearing upon Dr. Ley's well-known theory of laryngismus stridulus. It was noticed that animals after this injury would appear to be breathing with ease, till any struggle or other muscular exertion took place, when symptoms of suffocation immediately supervened, as if the stronger inspiratory action brought on the paroxysm, by producing so powerful a current of air as to close the passive lips of the glottis. From the experiments on the *cardiac branches* of the vagus, it may be inferred that although they convey mental and other impressions from the nervous centre to the heart, yet the movement of the latter organ may be affected by such causes after the division of the vagi, and recurrents in the neck. The observations upon the degree to which the action of the respiratory muscles was influenced by *division of the vagi* are very important: the movements uniformly became slower, though at first they were performed with ease; after a time they became more laborious; but some of the animals quite recovered the effects of the operation. The changes in the lungs were carefully noted in the animals which died asphyxiated, and Dr. Reid convinced himself that they could be sufficiently accounted for by the diminished frequency of the respirations, and the consequent congestion of the pulmonary vessels. Though there is good reason for believing that the impressions which excite the reflex respiratory action are chiefly conveyed to the medulla by the vagi, yet they appear to be also conveyed by the branches of the fifth pair distributed on the face. Dr. Reid wishing to observe whether the want of the *vagi* is compensated by volition, removed the cerebrum and cerebellum; but he found that the respiratory movements still continued, though greatly diminished in frequency. The observations on the *gastric* branches of the vagi were not less interesting. In several animals the muscular movements of the stomach continued, the gastric secretion returned, the appetite was restored, chyle was found in abundance in the thoracic duct, and, in fact, the whole digestive process was re-established, notwithstanding dissection proved that the operation had been performed most completely: at the same time it was proved that section of the nerves very materially deranges the digestion for a time, though if the animal live long enough, the effect will subside. It should be remarked, that in all the experiments on the vagi, Dr. Reid took care to maintain the entrance of air into the trachea. If the animal was not full-grown, the larynx being therefore less developed, a tube was introduced into the windpipe.

At the sitting of the Académie de Médecine, January 21, 1839, MM. Jules Guyot and Casalis presented an account of their experiments on the nerves of the tongue. The glosso-pharyngeal betrayed great sensibility when pricked, pulled, or cut: the division of it impeded deglutition, and appeared to destroy the perception of certain flavours, but not of all; probably those which are felt at the base of the tongue. Division of the lingual branch of the fifth pair destroyed the tactile sensibility and the sense of taste in the three anterior fourths of the tongue. Division of the hypoglossal paralysed the same portion of the tongue, but left the taste and tactile sensibility unimpaired.

One result of these experiments is opposed to the conclusion from those of Panizza as to the function of the lingual branch of the fifth pair. The latter observer considered that it imparts nothing more than tactile sensibility. The true opinion seems to be, that this nerve comprises some fibrils subservient to taste, and others to common sensation. This two-fold function is presumable from a striking case related by Dr. Romberg, in *Müller's Archives*, of paralysis of the third branch of the fifth pair on the left side, which was caused by pressure upon this nerve just after its issue from the cranium. In this case there was loss of taste as well as of tactile sensibility on the left half of the tongue. As for the glosso-pharyngæal, Dr. Romberg throws out the very probable idea that the signs of nausea and retching, caused by irritation of this nerve, have been mistaken for indications of taste.

---

AN ADDRESS UPON LAYING THE FOUNDATION-STONE OF THE QUEEN'S HOSPITAL, BIRMINGHAM, JUNE 18, 1840. WITH NOTES AND AN APPENDIX. By *Vaughan Thomas,* B.D. Vicar of Stoneleigh, Warwickshire, and formerly Fellow and Tutor of Corpus Christi College, Oxford. To which is prefixed, an Account of the Ceremony, and the List of Subscriptions to June 20. Printed at the Request of the Council of the Royal School of Medicine and Surgery. Oxford, 1840.

We are right glad to see two things of which this pamphlet offers us ample evidence—first, the powerful interest taken by the public in the erection of provincial hospitals and prosperity of provincial schools—and secondly, the zealous manner in which our able and excellent clergy are aiding the sacred cause of medical education. All this is very evident from the history of the Birmingham School of Medicine.

We are tempted to extract one passage from the pamphlet now before us. The eloquent and reverend Orator deplores the silence of the clergy in their addresses to the public sympathy and charity on the educational objects and value of hospitals. The benefit to the sick patient has been descanted on, but the wider benefit to the whole human race from the knowledge conferred on the student of medicine has been passed in utter silence. Let us hope for better things.

" I impute not," says Dr. Thomas, "these omissions to the worthy, the charitable, the munificent of those early days as personal faults; they are stated simply as facts; if they are to bear a harsher name, I would call them, not the faults of individuals, but of the age in which they lived; for some of them lived in days of darkness, and some of twilight, as to the great truths, that pathology must be studied like other natural sciences, under the guidance of facts and phænomena; that inductive philosophy, in this as in other applications of its power, demands instances and examples; that no safe or sure progress can be made in conducting the studies of the novice, or in completing those of the proficient, without the inspection and explanation of cases, without the power of referring to the different sorts of accidental or constitutional, of structural or functional mischief, as instanced in the sufferings of individuals; and where are these to be found in such contiguity to each other, in such variety and abundance, and under such diversities of internal or external character, as on the beds of an hospital? But to these incontestable truths, and to these wants of professional life; to the obvious interests of the public, and to the duties of charity in the dispensation of this necessary knowledge, founders, trustees, governors of hospitals, do not in early times appear to have paid any attention."

After pointing out what might have been urged, he goes on to say :—

" Such are some of the topics which a knowledge of the educational use and application of an Hospital would have administered to those, who, during more than two centuries and a half, have been successively called upon to plead the cause of St. Bartholomew's and St. Thomas's. With great power of language, and piety of purpose, and compass of scriptural warranties, have they urged the cause of poverty and sickness. But preparing the young for the care and cure of sickness, by teaching them what they ought to do in cases of accident or disease, and by giving them the manual, the moral, the intellectual ability to do it, were departments of charitable exertion, which in those days were neither understood by the advocates nor the administrators of hospitals. The merciful ends of such courses of medical and surgical education never entered into the calculations of the wise and good; it ought however to be added, in justice to those able and pious orators, that such topics, as the charitable offices of medicine and surgery, and the Christian duty of training up pupils and apprentices to be good practitioners by attending hospital practice, would scarcely have been understood in the general ignorance or indifference that prevailed, much less would they have been felt, as grounds and reasons for a more liberal contribution in those days of insensibility to the educational wants of the profession."

We are sure that the diffusion of such sentiments as these in the wide and influential ranks of the Church, would be productive of the utmost benefit.

---

The Maternal Management of Children, in Health and Disease. By *Thomas Bull*, M.D. Physician Accoucheur to the Finsbury Midwifery Institution, and Lecturer on Midwifery, and on the Diseases of Women and Children, &c. &c. London, 1840.

Dr. Bull informs us in his Preface that his book is written for the young mother. He tells us, too, that in the first chapters, devoted to the general management of the child in health, the author has endeavoured to teach the young mother, that *the prevention of disease is her province, not its cure;* that to this object all her best efforts must be directed; and, moreover, that to tamper with medicine, when disease has actually commenced, is to hazard the life of her offspring.

In the fourth chapter it has been attempted to point out, how the first symptoms of disease may be early detected by the parent. The subject has been felt to be a difficult one, and to give particular directions quite out of the question; but it is hoped that the suggestions thrown out will, in some measure, answer the purpose intended. On the advantage of an early and prompt application of remedies in the diseases of childhood, generally so active in their progress and severe in their character, it is unnecessary to offer any observation.

The latter part of the work, consisting of the maternal management of disease, the author regards as a subject of high and serious moment. Small as is the attention which has been hitherto paid to it, yet, in the diseases of infancy and childhood, how invaluable is a careful and judicious maternal superintendence to give effect to the measures prescribed by the physician.

We have looked over this little book, and we think it likely to be useful to the parties to whom it is avowedly addressed. The directions, though concise, are for the most part sufficiently full, and, are characterised by discretion and good sense.

---

Pharmacopée Universelle, ou Conspectus des Pharmacopées d'Amsterdam, Anvers, Dublin, Edimbourg, Ferrare, Genève, Grèce, Hambourg, Londres, Oldenbourg, Parme, Sleswig, Strasbourg, Turin, Wurzbourg; Americane, Autrichienne, Batave, Belge, Danoise, Espagnole, Finlandaise, Française, Hanovrienne, Hessoise, Polonaise, Portugaise, Prussienne, Russe, Sarde, Saxonne, Suedoise et Wurtembergeoise, &c. &c. By *A. J. L. Jourdan*, Membre de l'Académie Royale de Médecine. Second Edition. Paris, 1840.

M. Jourdan, in a sensibly written Preface, points out the necessity for an acquaintance with the various Pharmacopœiæ, and concludes, from the sale of his first edition, and the various translations of his works, that it has proved both acceptable and useful—and so, no doubt, it has done.

This new edition is, in fact, a new work. Whilst the old one presented the contents of 35 legal Pharmacopœiæ and 18 Formularies, this contains 42 Pharmacopœiæ and 31 Formularies. There is an account of the various weights and measures used in the different countries of Europe, and their relation to each other, borrowed from the admirable tables of Lehmann.

Of the execution of this edition we may speak from a cursory glance, in very high terms indeed. The work is one of very great value to every practical physician and surgeon, and no collection, even a moderate one, of useful works on medicine is complete without it. We recommend it, then, strongly to our readers, and still more strongly, if possible, to all book societies.

---

### Recent Works on Anatomy and Physiology.

### I. Quain's Plates—Fasciculi 50 to 70.

Let us not forget to chronicle these. They maintain (we think they do more) the character with which they started, and having now nearly reached completeness, they justify our reiterated recommendations as well as the purchaser's outlay. The nerves and viscera have been the subjects of the late Fasciculi. They are very well executed. We would, however, repeat the advice we have already given to Mr. Quain and his publisher, to add some supplementary Fasciculi, in order that the work may contain everything needful. No one will grumble at eight or ten extra numbers, if they lead to that. The only fault we find is one of this description.

### II. The Anatomist's Vade Mecum: a System of Human Anatomy. By *W. J. Erasmus Wilson.* With upwards of 150 Illustrations by Bagg. London, 1840.

This is a very good little book. Mr. Wilson observes:—"The student of medicine, from the first moment of commencing his labours in the study of anatomy, must be made aware of the absolute necessity that exists for clearness of thought, exactness of language, and a rigorous arrangement of ideas. He must feel confidence in the knowledge which he possesses, and he will then exhibit that confidence in the decision by which all his actions will be characterised. As a text-book for illustrating in a precise method the materials of instruction, this work is especially designed; and the severity and inflexibility of order have not been departed from in treating of a single branch of the subject."

"I hope I may be permitted to say that the engravings are beautiful examples of a most instructive and valuable art. The advantages of such illustrations in a demonstrative science cannot be too highly appreciated. The mode in which the engravings have been printed—a distinct branch of art in itself,—will not pass unnoticed by those who are acquainted with the complicated process and extreme care which are necessary to the production of the delicacy and force of effect of such graphic illustrations."

These illustrations are more than 150 in number. The wood-cuts are really exceedingly well executed, and singularly clear for their size. And they will afford much assistance to the student. We cordially recommend the Vade Mecum.

III. Elements of Physiology. Parts I. II. and III. Second Edition; Parts IV. and V. First Edition. By *J. Müller,* M.D. Translated by *William Baly,* M.D.

It is impossible to speak in too high terms of this admirable work. We rejoice to find that the sale is commensurate with the merits of it, and that the sound views, eminent sagacity, and excellent judgment of M. Müller are appreciated by the English Profession. It must form, as, indeed, it does, the text book of the Lecturer on Physiology, and to young and old, the student in the class room, or the practitioner in his library after the toils of the day, it will convey both useful and delightful knowledge. We commend it to all, and trust that by all it will be patronised.

We should state that the Parts already out contain:—

I. General Physiology—the Blood and Circulating System—the Lymph and Lymphatic System—Respiration—Nutrition, Growth, and Reproduction.

II. and III. Secretion, Digestion, Functions of the Glands without Efferent Ducts, Excretion, and the Nervous System.

IV. Ciliary Motion, Muscular and Allied Motion, Voice and Speech.

V. The Senses.

---

An Essay on the Treatment and Cure of Pulmonary Consumption, on Principles natural, rational, and successful. With Suggestions for an Improved Plan of Treatment of the Disease amongst the Lower Classes of Society; and a Relation of several successive Cases restored from the last Stage of Consumption to a good State of Health. By George Bodington, Surgeon. London, 1840.

It will soon appear that Mr. Bodington proposes a great alteration in our treatment of consumptive patients. He thinks, and perhaps he is not very wrong, that at present the complaint does us no great credit. That, we fear, will be generally admitted, whatever may be thought of his method.

The theory he entertains is easily set forth:—

"It will be observed, that the main ground of the treatment has been to preserve or restore to a normal condition, the functions of the nervous filaments, interwoven with the substance of the lungs, and exercising influence over the capillary system and other parts of the organization: it has been assumed that the first link in the chain of morbid actions arises there, as they first feel the irritation from the presence of the morbid matter deposited as a foreign body, and that all the other changes are consecutive to this wasting or destruction of the nervous energy of the filaments with which the tuberculous matter comes in contact. Upon this view the treatment of pulmonary consumption, in the way herein recommended, has been founded."

It seems to us that the presence of tubercles being granted, there is no need of the "wasting of the nervous energy" to explain the rest. Nor is there any proof of any such wasting at all. Disease has already advanced, the constitution is already contaminated, the first change of a series of changes has occurred when the tuberculous matter is laid down.

Whatever we may think of the theory, the practice founded on it is not bad, if the following statement is borne out by the result.

"An uniform and complete success having resulted in the treatment of several cases of tuberculous consumption, upon the principles and plan explained in the following pages, the author deems it his duty to publish them, with his opinions and principles of treatment."

Mr. Bodington is an enemy to confinement to the chamber—to tartarized antimony—to digitalis—to "demulcents, blisters, leeches, plaisters," &c.—to the sea-coast—to the inhalation of gases. The gas he likes is the fresh air, and it must be owned that it is after all a very good gas.

"The only gas fit for the lungs is the pure atmosphere freely administered, without fear; its privation is the most constant and frequent cause of the progress of the disease. To live in and breathe freely the open air, without being deterred by the wind or weather, is one important and essential remedy in arresting its progress;—one about which there appears to have generally prevailed, a groundless alarm lest the consumptive patient should take cold."

Now, then, for the plan.

"In order then to restore a consumptive patient, it will be necessary especially to attend to the following matters. We shall find first of all a rapid and weak pulse, ranging from 120 to 140 beats in a minute, clearly indicating a deficient supply of blood, and the heart and arteries irritable in proportion to this deficiency. This condition must be met at once, not by the means termed 'antiphlogistic,' but with frequent supplies, in moderate quantities, of nourishing diet and wine; a glass of good sherry or Madeira in the forenoon, with an egg, another glass of wine after dinner, fresh meat for dinner, some nourishing food for supper, such as sago, or boiled milk, according to the taste and digestive powers of the patient. This will be supplying means to rectify the morbid condition of the nutritive functions, and to allay the irritability of the heart and arteries. I have generally succeeded in the course of a few days, or perhaps a week, in reducing the pulse from 130 or 140 down to 90, by means of this diet, and by a systematic use of sedative medicines, and other means. The whole nervous system is unduly excited, or affected in some way we know not how to express or understand, from our limited knowledge of it, when under the influence of this disease, and neither can nutrition be affected, or the muscular system recover strengh, or the vessels be filled with a due supply of the vital fluid, unless that nervous disorder be allayed and soothed, or rendered more in accordance with a healthy condition. The plan to obtain this object is, to give alterative doses of sedatives, and also direct or full ones. The former consist of moderate doses given at intervals throughout the day, with the view of allaying the general nervous excitement. The direct or full dose is given at bed-time, to allay coughing and procure sleep. Aconite, henbane, or the salts of morphia may be used. I have preferred generally the hydrochlorate of morphine: A sufficient dose to procure a whole night's repose should be given every night, in addition to the alterative doses above-mentioned; the latter may be administered, in an almond emulsion, in doses repeated three or four times a day. Should the medicine produce constitutional effects, paleness, faintness, sickness, giddiness, it must be laid aside for a period, and an antidote will be found in small quantities of weak brandy and water, or wine and water. The sedative medicines should be resumed so soon as these effects are removed.

I come now to the most important remedial agent in the cure of consumption, that of the free use of a pure atmosphere; not the impure air of a close room, or even that of the house generally, but the air out of doors, early in the morning, either by riding or walking; the latter when the patients are able, but generally they are unable to continue sufficiently long in the open air on foot, therefore riding or carriage exercise should be employed for several hours daily, with intervals of walking as much as the strength will allow of, gradually increasing the length of the walk until it can be maintained easily several hours every day. The abode of the patient should be in an airy house in the country; if on an eminence the better: The neighbourhood chosen should be dry and high; the soil, generally of a light loam, a sandy or gravelly bottom; the atmosphere is in such situations comparatively free from fogs and dampness. The patient ought never to be deterred by the state of the weather from exercise in the open air; if wet and rainy, a covered vehicle should be employed, with open windows. The cold is never too severe for the consumptive patient in this climate; the cooler the air which passes into the lungs, the greater will be the benefit the patient will derive. Sharp frosty days in the winter season are most favourable."

We dare say Mr. Bodington will not admire our opinion, or deem it the most enlightened in the world. Yet, such as it is, we give it. We are really disposed to go some way with him, though we cannot go the whole. We quite agree with him that to treat consumption by good doses of antimony, digitalis, and drugs of that sort, is neither consistent with sound pathology, nor attended with anything like success. We agree with him too, that there are many cases, incipient ones more particularly, where the open air, judiciously indulged in, is highly valuable, and infinitely preferable to confinement. But we cannot subscribe to the general use of wine, and to the selection of elevated situations for residence, and to the exposure to weather, and to the proscription of regulated confinement and of counter-irritation, in all cases of phthisis. In this, as in other things, there is a mean which a sound judgment will seek after and maintain. And, so far as our experience has gone, great benefit has accrued from the careful employment of measures of this very sort. Yet we do think they have been abused, and, although there is something bizarre in Mr. Bodington's hypothesis, and extreme in the treatment he has founded on it, this very extravagance (if such there be) may awaken useful reflections in the minds of such as commit extravagances on the other side.

---

### CUTANEOUS DISEASES.

I. Commentaries on Diseases of the Skin. By *Anthony Todd Thomson*, M.D. &c. &c. &c. Illustrated by Coloured Plates, representing the Commencement, Progress, and Termination of the Eruptions,—Drawn, Lithographed, and Coloured by Joseph Perry. Taylor and Walton, London, 1840.

Three Fasciculi, with the Atlas of each, lie before us. The two first are devoted to the Scaly Eruptions, the third to Herpes and Rupia. We shall notice the letter-press more fully next quarter. It only remains for us to speak in very favourable terms of it now, and to refer to the plates. To say that they are executed by Mr. Perry is to say almost enough. They exhibit all his fidelity of drawing, his finish of execution, his brilliancy (shall we say too great) of colouring. But we shall revert to the work next quarter, and examine it more in detail.

II. Illustrations of Cutaneous Disease, &c. By *Robert Willis*, M.D. Parts XVII. XVIII. and XIX. for July, August, and September.

Fresh faces must not drive away our recollection of an old friend. Dr. Willis's excellent work continues, and it fully maintains its reputation. We have vividly depicted Herpes Circinnatus—Hypertrophia Folliculorum—Rupia Prominens—Lichen Acutus and Lichen Chronicus—Prurigo—Acné—Acné Rosacea (to the life)—Chloasma—Trichosis Menti—Eczema Chronicum Pudendorum—Anthrax—Elephantiasis Arabica.

Of all we can speak in high terms, and of each we shall speak more at large next quarter, when we shall bring together the works of Dr. Willis and of Dr. AT. Thomson.

### ZOOLOGY AND NATURAL HISTORY.

I. A GENERAL OUTLINE OF THE ANIMAL KINGDOM AND MANUAL OF COMPARATIVE ANATOMY. By *Thomas Rymer Jones*, F.Z.S. Professor of Comparative Anatomy in King's College, London. Illustrated by numerous Engravings on Wood. Parts X. and XI. Price 2s. 6d. each Part. Van Voorst, London, 1840.

Mr. Jones has arrived at the Vertebrata, and Fishes are served up in the eleventh Part before us. The execution of the work does not flag, no trifling encomium, and the mechanical execution of both letter-press and wood-cuts is admirable. We would reiterate our recommendations to our readers to possess this work.

II. A HISTORY OF BRITISH BIRDS. By *William Yarrell*, V.P.Z.S. Illustrated by a Woodcut of each Species and numerous Vignettes. Parts XVIII. and XIX. Van Voorst, London, 1840.

Mr. Yarrell is again here with his delightful sketches of our Native Birds. And a pretty looking volume they will make. The work, we see, verges towards its completion. Had we any *fair* readers we would press it on them; the doctor's drawing-room table should be furnished with it, even though the shades of Hippocrates should frown it from the sacred shelf devoted to the sad and learned writers on our science.

---

THE NATURALIST'S LIBRARY. Conducted by Sir *Wm. Jardine*, Bart. Ichthyology; treating of the Nature, Structure, and Economical Uses of Fishes. By *J. S. Bushnan*, M.D. pp. 219, with numerous plates. Lizars, Edinburgh. Highley, London, 1840.

In this most interesting volume, Dr. Bushnan has presented us with a highly characteristic portrait of the habitats, anatomy, physiology, and economy of that great class of animated beings which inhabit the "waters under the earth" —which existed perhaps millions of years before MAN was created—and which forms a connecting link between the beasts of the field and the birds of the air. The work is executed with singular care and ability, and does Dr. Bushnan infinite credit. It is preceded by a memoir of Hippolito Salviani, an author probably unknown, even by name, to the majority of our readers, but who cultivated natural history in general, and ichthyology in particular, in a distant age, in troublesome times, and with praiseworthy assiduity. Baron Cuvier and our present author have rescued the venerable name of Salviani from comparative, but unmerited obscurity, and deserve the thanks of all the lovers of God's wonders on this globe. The plates of this volume are admirably executed and beautifully coloured. One other volume on the same subject is promised from the pen of that able and enterprizing traveller, M. Schomburgh, "*On the Fishes of the Essequibo, and the Rivers of British Guiana.*" We shall hail it with great pleasure; in the mean time we return our best thanks to the talented author of the present volume, and wish him long life and that greatest of blessings— GOOD HEALTH.

